# How Important Is ‘Accuracy’ of Surrogate Decision-Making for Research Participation?

**DOI:** 10.1371/journal.pone.0054790

**Published:** 2013-01-31

**Authors:** Scott Y. H. Kim, H. Myra Kim, Kerry A. Ryan, Paul S. Appelbaum, David S. Knopman, Laura Damschroder, Raymond De Vries

**Affiliations:** 1 Center for Bioethics and Social Sciences in Medicine, University of Michigan, Ann Arbor, Michigan, United States of America; 2 Department of Psychiatry, University of Michigan, Ann Arbor, Michigan, United States of America; 3 Center for Statistical Consultation and Research, University of Michigan, Ann Arbor, Michigan, United States of America; 4 Division of Law, Ethics, and Psychiatry, Department of Psychiatry, Columbia University and New York State Psychiatric Institute, New York, New York, United States of America; 5 Department of Neurology, Mayo Clinic, Rochester, Minnesota, United States of America; 6 Health Services Research and Development, Ann Arbor VA Medical Center, Ann Arbor, Michigan, United States of America; University of São Paulo, Brazil

## Abstract

**Background:**

There is a longstanding concern about the accuracy of surrogate consent in representing the health care and research preferences of those who lose their ability to decide for themselves. We sought informed, deliberative views of the older general public (≥50 years old) regarding their willingness to participate in dementia research and to grant leeway to future surrogates to choose an option contrary to their stated wishes.

**Methodology/Principal Findings:**

503 persons aged 50+ recruited by random digit dialing were randomly assigned to one of three groups: deliberation, education, or control. The deliberation group attended an all-day education/peer deliberation session; the education group received written information only. Participants were surveyed at baseline, after the deliberation session (or equivalent time), and one month after the session, regarding their willingness to participate in dementia research and to give leeway to surrogates, regarding studies of varying risk-benefit profiles (a lumbar puncture study, a drug randomized controlled trial, a vaccine randomized controlled trial, and an early phase gene transfer trial). At baseline, 48% (gene transfer scenario) to 92% (drug RCT) were willing to participate in future dementia research. A majority of respondents (57–71% depending on scenario) were willing to give leeway to future surrogate decision-makers. Democratic deliberation increased willingness to participate in all scenarios, to grant leeway in 3 of 4 scenarios (lumbar puncture, vaccine, and gene transfer), and to enroll loved ones in research in all scenarios. On average, respondents were more willing to volunteer themselves for research than to enroll their loved ones.

**Conclusions/Significance:**

Most people were willing to grant leeway to their surrogates, and this willingness was either sustained or increased after democratic deliberation, suggesting that the attitude toward leeway is a reliable opinion. Eliciting a person’s current preferences about future research participation should also involve eliciting his or her leeway preferences.

## Introduction

Alzheimer’s disease (AD) is incurable, devastating, and highly prevalent. The clinical research necessary to make progress against AD, however, poses the dilemma of involving persons lacking the capacity to provide informed consent. Although some persons with mild AD may be able to provide consent, the disease leads to early decisional incapacity [1], [2], [3], and surrogate consent for research is usually necessary. But surrogate consent may be seen as problematic–despite broad support for the practice of surrogate consent for dementia research among the older general public and caregivers of persons with AD [4], [5], [6]–because surrogates’ judgments about their loved ones’ preferences, both in the clinical [7], [8] and research settings [9], [10], are often discordant. Further, although surrogates might be helped by prior communication of research participation preferences by their loved ones, most people fail to communicate such preferences, even when they say they will do so [11].

Pessimism about the accuracy of surrogate consent, however, may need to be tempered by the evidence that most people do not seem to value the concordance between their current preferences and their future surrogates’ decisions as much as one might assume. Studies have found that even when subjects voice a preference regarding treatment [12], [13], [14] or research involvement [5], [11], [15], [16] in the future, most are willing to grant at least some leeway to their surrogates to override that stated preference. However, most of these reports on persons’ willingness to grant leeway in decision making regarding research are based on traditional surveys (of selected populations from clinics, senior centers, or ongoing research studies), in which the complex scientific, legal, historical, and bioethical dimensions of the ethics of dementia research–which are unfamiliar to most laypersons–are not fully explained to the respondents. Given perennial concerns about the concordance between a person’s current preferences and future decisions by his or her surrogate and the implications of leeway preferences for those concerns, optimizing the internal validity of studies on respondents’ preferences regarding leeway is important. In this paper, we report what happens to people’s preferences regarding participation in dementia research and their views on leeway when they receive in-depth, balanced information and deliberate with peers in a day-long session dedicated to the issue.

This report is part of a larger study whose primary focus was on the impact of democratic deliberation on the older (≥50 years old) general public’s preferences regarding societal policy for addressing research with decisionally incapable elderly persons [6]. Here we report the effect of deliberation on the older general public’s willingness to participate in dementia research and to give leeway to future surrogate decision-makers, i.e., their attitudes regarding surrogate consent for dementia research from the perspective of potential participants. We examine the predictors of willingness to give at least some leeway to surrogates. In addition, we assessed respondents’ willingness, as potential surrogates, to enroll a loved one in research.

## Methods

A detailed account of the theoretical basis and methodological procedures for this study is available at http://tinyurl.com/DD-methods-PDF [17], [18].

### Participants

Members of the older general public (≥50 years old) within a 50-mile radius of Ann Arbor, Michigan were recruited through the Survey Research Center of the Institute for Social Research (ISR) at the University of Michigan via random-digit dialing (recruitment flowchart available at http://tinyurl.com/DD-FlowChart). Of 2402 eligible individuals contacted, 700 (29%) agreed to participate in the study; of these, 503 (72%) completed the baseline survey (Survey 1) and were randomized to one of three groups: 212 to a deliberative session group, 141 to an education group, and 150 to a control group. Of those assigned to the deliberative session, 160 (75%) attended an all-day education and deliberation session. The education only group received by mail the annotated slide presentations of the experts used in the deliberative session (available at http://tinyurl.com/DD-Presentations). The control group received surveys only, without any interventions.

### Ethics Statement

This study was deemed exempt from review under the federal regulations on human subjects research by the University of Michigan’s Institutional Review Board.

### Measures

The survey administered to subjects has been used in previous studies to assess several aspects of attitudes toward surrogate consent for dementia research [4], [17]. It contains an introduction to Alzheimer’s disease and to the ethical dilemma of involving decisionally impaired subjects in research, and presents four scenarios of approximately 120 words each describing hypothetical research studies. The four scenarios depict: a study to develop a diagnostic test requiring a lumbar puncture; a randomized controlled trial for a new drug; a randomized controlled trial of an AD vaccine; and an early-phase neurosurgical gene-transfer trial.

In this paper, we focus on the following questions from the survey: “Suppose you wanted to give a close family member instructions for the future, in case you ever became unable to make decisions for yourself. Would you say you would want to participate in the study?” (We will refer to this as the ‘self-perspective’ question). The response options were: definitely yes, probably yes, probably not, and definitely not. The self-perspective question was followed up with: “How much freedom or leeway would you give the close family member to go against your preference, and instead [opposite of stated preference: enroll/not enroll you in the study]?” (leeway question) with response options of no leeway, some leeway, or complete leeway. For how subjects would choose if they were themselves acting as surrogates, we asked: “Suppose you have a loved one who has Alzheimer’s disease and cannot make decisions for him or herself. Would you give permission for your loved one to be part of this study?” (We will refer to this as the ‘surrogate perspective’ question). The survey was written at an 8^th^-grade reading level (Flesch-Kincaid grade level 8.4). For each question, subjects had an opportunity to provide comments.

The survey was administered to each subject three times. Survey 1 was administered by mail prior to randomization, about one month before the deliberation date. Survey 2 was completed at the end of the deliberation day (for the deliberation session attendees) or around that date (by mail, for all others). Copies of the deliberation day presentations (slides plus notes in PowerPoint®) were mailed to the education group with Survey 2. Survey 3 was sent by mail approximately 1 month after the deliberation date.

### Deliberation Session

On the day of the deliberation session, the attendees were randomly assigned to tables, in groups of 5–7 persons per table. The subjects were educated by experts in clinical research and in bioethics, during a plenary session, using two 45-minute presentations (followed by 15 minutes of Q&A): “Alzheimer’s Disease Clinical Research” described features of Alzheimer’s disease, current treatment, types of research on AD and treatment development process, and how subjects are enrolled in research; “Ethical Issues in Surrogate-Based Research” described well-known human subject abuses (including the Nazi experiments, Tuskegee study, and Willowbrook), the resulting regulatory process, the unsettled policy on surrogate consent, and the reasons for and against surrogate consent for dementia research [17]. In developing these presentations, the research team worked closely with an advisory panel consisting of a political science expert in deliberative democracy methods, a senior AD researcher, a bioethicist-sociologist, a geriatrician, a director of a human subject protections program at an academic medical center, a qualitative research expert, a gerontological nurse, and a caregiver of a person with AD. These presentations were further refined, based on a final systematic review by the members of the advisory panel, additional external experts (in both AD research and bioethics), and laypersons [17]. Throughout the day, the subjects had multiple opportunities to question the experts, and engaged in three small-group deliberation sessions, moderated by a trained facilitator [6].

### Analyses

The subjects’ responses to the three main questions [self-perspective (willingness to participate), leeway (willingness to grant leeway), and surrogate perspective (willingness to enroll a loved one)] were compared across the three study groups to assess the effects of democratic deliberation (DD) and education (i.e., via written information) relative to the control condition. The analyses were conducted separately for each of the 4 scenarios for each study question. We used a generalized linear mixed-effects model with subjects as random intercepts to adjust for within-subject correlation in responses. The dependent variable was the *change* in responses to the three main study questions, based on changes from baseline (Survey 1, one month prior to DD session date) in the responses at Survey 2 (just after DD session for DD group, around that date for the rest) and at Survey 3 (approximately 1 month after DD session date). This variable’s value reflects changes in the 4-point scale (definitely yes, probably yes, probably no, definitely no) with a range of −3 to +3 for self-perspective and surrogate perspective responses and changes in the 3-point scale with a range from −2 to +2 for the leeway question (none, some leeway, complete leeway), with positive changes indicating greater willingness.

Each model included baseline values of the response, an indicator of DD group at Survey 2 (i.e., an interaction of DD group at Survey 2) and an indicator of education group at Survey 2 (i.e., an interaction of education group by Survey 2) to test for DD and education effects relative to control group at Survey 2. The model also included an indicator for Survey 3 for time effect at one month after the DD session in the control group, an indicator of DD group at Survey 3 to test for the DD effect relative to control group at Survey 3, and similarly an indicator for education group at Survey 3 to test for the education effect relative to control group at Survey 3. The difference between DD and education effects at each survey time was compared based on the model as post-hoc tests. All analyses involving the deliberation group conservatively included all those assigned to the deliberation group (i.e., both deliberation session attendees and non-attendees).

In addition, for each of the 4 scenarios, differences in responses to the “willingness to participate” (self-perspective) and “willingness to enroll a loved one” (surrogate perspective) questions were compared across the study groups and assessment times using a generalized linear mixed effects model with the differences in responses to the two questions as the dependent variable, with subjects as random intercepts.

Lastly, we evaluated the predictors of willingness to give leeway by using the baseline data (Survey 1) for the entire sample. For this analysis, we constructed logistic regression models by dichotomizing the willingness to give leeway response (as complete/some leeway vs. no leeway) because we wanted to assess the difference, if any, between those who would not grant any leeway at all versus others who would at least consider some leeway. The potential predictors examined were gender, race, education level, financial status, relationship to AD patient, marital status, and age, along with responses from three different perspectives (responses to the self-perspective and surrogate-perspective questions above, as well as to the societal perspective question, “If patients cannot make their own decisions about being in studies like this one, should our society allow their families to make the decision in their place?”) as dichotomized variables (definitely/probably yes versus definitely/probably no).

We also examined qualitative comments on the leeway question for all three surveys. In all, there were 716 comments representing 222 different respondents. We grouped the comments by participant “willingness to participate” (self-perspective dichotomized as definitely or probably not willing vs. definitely or probably willing) and “willingness to give leeway” (dichotomized as no leeway vs. some/complete leeway). Two team members (SK, KAR) independently read the comments to generate core themes and categories. This coding scheme was iteratively refined by using feedback from three coders (KAR and 2 research assistants). The comments were coded using the final coding scheme by consensus among the three coders. In the few instances where consensus could not be reached (less than 1% of comments), the decision was made by majority vote.

## Results

Subject characteristics are summarized in Table 1. There were no differences across the three arms (DD group, education-only group, control group) in any of the participant characteristics measured [6].

**Table 1:** Participant characteristics at randomization, N = 503. (This table used with permission [6].)

	DD (N = 212)	Education (N = 141)	Control (N = 150)	p-value^a^
Female	127 (60)	94 (67)	96 (64)	0.42
Age in years (mean (SD))^b^	63 (8)	63 (9)	63 (10)	0.81
Marital status				
Single	20 (10)	12 (9)	18 (12)	
Married	132 (63)	87 (62)	85 (57)	
Divorced	37 (18)	24 (17)	26 (17)	0.86
Widowed	21 (10)	18 (13)	20 (13)	
Other	1 (1)	0 (0)	0 (0)	
Hispanic or Latino/Latina	1 (1)	1 (1)	1 (1)	0.96
Race				
White	183 (86)	125 (89)	127 (85)	
Black or African-American	25 (12)	14 (10)	21 (14)	0.72
Other	4 (2)	2 (1)	1 (1)	
Highest level of education				
Less than BA	110 (52)	85 (60)	78 (52)	
BA	54 (26)	35 (25)	41 (27)	0.39
More than BA	48 (23)	21 (15)	31 (21)	
Finances at the end of a typical month^c^				
Some money left over	137 (65)	91 (65)	98 (65)	
Just enough to make ends meet	54 (26)	35 (25)	35 (23)	0.99
Not enough to make ends meet	12 (6)	9 (6)	8 (5)	
Relationship with an Alzheimer’s patient				
Primary Caregiver/Decision-maker	56 (27)	41 (30)	34 (23)	
Close to someone with AD	90 (43)	61 (44)	66 (44)	0.66
No relation	65 (31)	37 (27)	50 (33)	

a Based on χ^2^ tests, except for age which is based on ANOVA.

b All cell values are N(%) except for age.

c Some percentages do not add to 100 because not all participants answered the question.

### Baseline

For the overall sample at baseline (n = 503), 84% of subjects responded that they were willing (41.2% definitely and 42.3% probably) to participate in the lumbar puncture study, 92% in the new drug RCT (48.9% definitely and 43.5% probably), 55% in the vaccine study (16.7% definitely and 38.0% probably), and 48% in the gene transfer study (11.9% definitely and 35.6% probably) (See Figure S1). A majority of respondents at baseline were willing to give some or complete leeway to a close family member to go against their currently stated preferences regarding future research participation: 69% for the lumbar puncture study (54.5% some leeway and 14.5% complete leeway), 71% for the new drug RCT (55.1% some, 15.5% complete), 61% for the vaccine study (52.3% some, 9.1% complete), and 57% for the gene transfer study (47.3% some, 9.7% complete). For those who were willing to participate in research, 74–81% were willing to give leeway to a family member. Even among those who were not willing to participate in future research, 36–43% of respondents were still willing to give some or complete leeway to family members to enroll them, depending on the scenario.

The responses to all three willingness questions for all three groups are presented in Tables S1 and S2. There were no differences in the distribution of responses at baseline across the three groups, except for the self-perspective response to the new drug study scenario (χ^2^ test, p = 0.04).

### Attitudes Toward Participation in Research and Leeway

Table 2 gives the DD and education effect on attitudes toward participating in research (self-perspective) for each scenario, estimated using a linear mixed-effects model based on changes in responses at Survey 2 and Survey 3 from Survey 1 (See Table S1 for corresponding summary frequency data).

**Table 2:** Linear mixed-effects model results of changes in responses to self-perspective question from Survey 1 (baseline), adjusted for baseline responses.

Variables	Lumbar Puncture			New Drug RCT			Vaccine			Gene Transfer		
	Beta	95% CI	p-value	Beta	95% CI	p-value	Beta	95% CI	p-value	Beta	95% CI	p-value
Survey 2*DD^a^	**.30**	**.17,.44**	**0.000**	**.23**	**.10, .36**	**0.001**	**.28**	**.10, .46**	**0.003**	**.21**	**.02, .39**	**0.029**
Survey 2*Education^a^	.01	−.13,.15	0.888	−.03	−.17,.11	0.688	.08	−.11,.27	0.420	.17	−.03, .36	0.102
Survey 3^b^	−.08	−.19,.02	0.107	−.01	−.10,.09	0.887	.11	−.02,.24	0.086	.04	−.09, .18	0.550
Survey 3*DD^c^	**.26**	**.13,.40**	**0.000**	**.19**	**.06, .32**	**0.004**	**.21**	**.03, .39**	**0.020**	**.27**	**.09, .46**	**0.004**
Survey 3*Education^c^	−.01	−.16,.13	0.846	−.07	−.21, .06	0.287	−.12	−.31, .07	0.232	.01	−.19, .21	0.941

a Effect of democratic deliberation (DD) or education relative to control group at Survey 2 on attitudes toward participating in research (self-perspective).

b Effect of Survey 3 to Survey 2 in control group (i.e., time effect) on attitudes toward participating in research (self-perspective).

c Effect of democratic deliberation (DD) or education relative to control group at Survey 3 on attitudes toward participating in research (self-perspective).

No education effect, compared with control group, was found in the willingness to participate from self-perspective. However, in each of four scenarios, there was a significant increase in willingness to participate in the DD group immediately after the deliberation compared to control group, and this change was sustained after a month, as shown by a significant Survey 3 by DD group interaction. For example, for the gene transfer scenario, the DD effect compared to control group at Survey 2 was 0.21 (p = 0.03), and at Survey 3, the DD effect was even higher (beta = 0.27; p = 0.004) compared with control group. Comparing DD group versus education group parameter estimates showed a difference for all scenarios except for the gene transfer scenario (p<0.001 for lumbar puncture and new drug scenarios, p = 0.04 for vaccine scenario, p = 0.67 for gene therapy scenario). One month later, the DD effect remained significantly higher when compared with the education group for all four scenarios (p<0.001 for lumbar puncture, drug RCT, and vaccine scenarios and p = 0.006 for gene transfer scenario).

For the leeway question (Table 3), the DD group became more willing to give leeway for the lumbar puncture scenario (beta = 0.15; p = 0.024) and for the vaccine scenario (beta = 0.17; p = 0.016) after the deliberation session at Survey 2, and this effect was sustained at 1 month for the lumbar puncture scenario.

**Table 3:** Linear mixed-effects model results of changes in responses to leeway question from Survey 1 (baseline), adjusted for baseline responses.

Variables	Lumbar Puncture			New Drug RCT			Vaccine			Gene Transfer		
	Beta	95% CI	p-value	Beta	95% CI	p-value	Beta	95% CI	p-value	Beta	95% CI	p-value
Survey 2*DD^a^	**.15**	**.02, .29**	**0.024**	.02	−.11, .15	0.793	**.17**	**.03, .31**	**0.016**	.05	−.09, .19	0.479
Survey 2*Education^a^	.07	−.07, .21	0.331	.00	−.14, .15	0.965	.08	−.07, .23	0.279	.02	−.13, .17	0.760
Survey 3^b^	−.04	−.14, .07	0.478	−.05	−.15, .06	0.406	**.11**	**.01, .21**	**0.036**	.00	−.11, .11	0.993
Survey 3*DD^c^	**.15**	**.01, .28**	**0.030**	.07	−.06, .21	0.277	.08	−.05, .22	0.239	**.19**	**.05, .33**	**0.007**
Survey 3*Education^c^	.02	−.13, .16	0.814	−.02	−.16, .13	0.829	−.09	−.23, .06	0.251	.02	−.13, .17	0.797

a Effect of democratic deliberation (DD) or education relative to control group at Survey 2 on attitudes toward giving leeway to family member in making research decisions if respondent is unable to make decision for self.

b Effect of Survey 3 to Survey 2 in control group (i.e., time effect) on attitudes toward giving leeway to family member in making research decisions if respondent is unable to make decision for self.

c Effect of democratic deliberation (DD) or education relative to control group at Survey 3 on attitudes toward giving leeway to family member in making research decisions if respondent is unable to make decision for self.

The education group was not different from the control group at either time point for any of the scenarios. The DD effects for the lumbar puncture and vaccine scenarios at Survey 2 were not significantly different when compared with the education group. On the other hand, for the gene transfer study, while there was no DD effect on leeway at Survey 2, the DD group expressed greater willingness to give leeway after 1 month compared to the control group (p = 0.007), and this delayed DD effect was significant even when compared with the education group (p = 0.02).

### Qualitative Responses

Among those who said they *would* give leeway (regardless of whether they would be willing to participate or not), the main reason they provided was that the surrogates would have more or better information in the future: “There could be new info that needs to be factored into decision making process” (Subject#353) and “They could have new information that wasn’t available when I gave instructions for the future” (S#266). For those who opted to give leeway but had said “no” to participating in the study, they noted in addition that the ratio of the risks/burdens vs. benefits may be different at the time of the study or that the surrogates may be able to better assess the risks at the time: “Possibly in the future a safer and more effective vaccine will be developed” (S#312) and “Based on new finding for benefits & reduced risk” (S#668).

Those who would *not* give leeway tended to see leeway as something that violated their right to make decisions for themselves: “I feel very strongly that the choices I make about my fate are mine and should not be changed”(S#131). Those who said “no” to both research participation and to leeway also frequently emphasized the risk/burden or lack of direct benefit of research as a reason for not allowing leeway. For example, one participant commented regarding the vaccine scenario: “The brain inflammation is the show stopper for me. While probably severe at any age, I see it as life threatening for the elderly. It’s one thing going into this trial without knowing particular risks. It’s totally unacceptable to knowingly put people at risk” (S#016).

### Attitude Toward Acting as a Surrogate

Table 4 presents the effect of DD and of education on willingness to enroll a loved one in future research (surrogate perspective) for each scenario at Survey 2 and at Survey 3. (The corresponding summary data are presented in Table S2.).

**Table 4:** Linear mixed-effects model results of changes in willingness to give permission for a loved one to participate in research (surrogate perspective) from Survey 1 (baseline), adjusted for baseline responses.

Variables	Lumbar Puncture			New Drug RCT			Vaccine			Gene Transfer		
	Beta	95% CI	p-value	Beta	95% CI	p-value	Beta	95% CI	p-value	Beta	95% CI	p-value
Survey 2*DD^a^	**.43**	**.29, .57**	**0.000**	**.29**	**.16, .42**	**0.000**	**.34**	**.17, .51**	**0.000**	**.19**	**.01, .36**	**0.038**
Survey 2*Education^a^	.10	−.05, .26	0.188	.08	−.06, .22	0.264	**.20**	**.01, .38**	**0.036**	**.25**	**.06, .43**	**0.010**
Survey 3^b^	.01	−.10, .12	0.904	.03	−.07, .14	0.523	.11	−.01, .24	0.066	.02	−.11, .15	0.723
Survey 3*DD^c^	**.21**	**.07, .35**	**0.004**	**.14**	**.01, .27**	**0.041**	**.21**	**.05, .38**	**0.013**	**.28**	**.10, .45**	**0.002**
Survey 3*Education^c^	−.05	−.21, .10	0.500	−.02	−.16, .12	0.791	−.06	−.24, .12	0.503	.09	−.10, .28	0.346

a Effect of democratic deliberation (DD) or education relative to control group at Survey 2 on willingness to give permission for a loved to participate in research.

b Effect of Survey 3 to Survey 2 in control group (i.e., time effect) on willingness to give permission for a loved to participate in research.

c Effect of democratic deliberation (DD) or education relative to control group at Survey 3 on willingness to give permission for a loved to participate in research.

Compared with the control group, the deliberation group became more willing to enroll a loved one in research for all four scenarios after the deliberation session, but the DD effect was not significant for vaccine and gene therapy scenarios when compared with the education group. On the other hand, changes in surrogate perspective willingness were sustained 1 month later in all four scenarios. Although the effect sizes were notably smaller after one month compared with the effect size immediately after the deliberation for the lumbar puncture, new drug and vaccine study scenarios, the DD effect at one month was significant for all four scenarios when compared with both control and education groups. In the case of the education group, significant initial changes (at Survey 2) were seen in their willingness to enroll a loved one in the vaccine study and the gene transfer study; however, these changes were not present after a month.

Respondents were more likely to participate themselves (self-perspective) than to be willing to enroll a loved one (surrogate perspective) for each of the four scenarios and across all three time points. Averaged across the three survey times and across the three study groups on the 4 point scale (definitely yes, probably yes, probably no, definitely no) respondents were more willing to participate themselves than to enroll a loved one for lumbar puncture study by 0.27 points (p<0.001) for the lumbar puncture study, by 0.22 points (p<0.001) for the new drug study, by 0.17 points (p<0.001) for vaccine study and by 0.18 points (p<0.001) for gene transfer study.

### Predictors of Leeway

At baseline (Survey 1), those willing to participate in future research themselves were more willing to grant leeway to surrogate decision-makers, even after controlling for willingness to allow a societal policy of family surrogate consent and other participant characteristics, for three scenarios (lumbar puncture, OR = 2.75, p = 0.01; vaccine RCT, OR = 3.55, p<0.001; gene transfer, OR = 5.03, p<0.001, Table 5). Those who would allow a societal policy of surrogate consent were more willing to grant leeway for all scenarios (lumbar puncture, OR = 2.05, p = 0.04; drug RCT, OR = 3.40, p = 0.005; vaccine RCT, OR = 2.37, p = 0.001; gene transfer, OR = 3.87, p<0.001). Increasing age predicted willingness to give leeway, but only in the two lower-risk scenarios (lumbar puncture OR = 1.03, p = 0.03; Drug RCT, OR = 1.03, p = 0.02). Those with greater than a bachelor’s degree were less willing to give leeway than those with less than a bachelor’s degree for the gene transfer scenario. Gender, race, financial status, relationship to AD patient, marital status, and surrogate-perspective responses were not associated with willingness to allow leeway.

**Table 5:** Predictors of leeway responses at baseline.

Variables	Lumbar Puncture			New Drug			Vaccine			Gene Transfer		
	OR	p-value	95% CI	OR	p-value	95% CI	OR	p-value	95% CI	OR	p-value	95% CI
Education level:												
Bachelor’s Degree	.69	0.139	.42–1.13	.81	0.415	.49–1.34	.80	0.389	.49–1.32	1.14	0.623	.68–1.89
>Bachelor’s Degree	.77	0.341	.45–1.32	.92	0.774	.53–1.61	.79	0.404	.46–1.37	**.49**	**0.014**	**.28–.86**
Age	**1.03**	**0.032**	**1.00–1.05**	**1.03**	**0.022**	**1.00–1.05**	1.00	0.929	.98–1.02	1.00	0.720	.97–1.02
Self-perspective	**2.75**	**0.011**	**1.26–6.00**	1.90	0.205	.71–5.09	3.55	**0.000**	**1.81–6.94**	**5.03**	**0.000**	**2.50–10.13**
Societal perspective	**2.05**	**0.037**	**1.04–4.04**	**3.40**	**0.005**	**1.46–7.91**	**2.37**	**0.001**	**1.42–3.94**	**3.87**	**0.000**	**2.24–6.67**

Note: The model was also adjusted for gender, race, financial status, relationship to an Alzheimer’s patient, surrogate perspective response, and marital status. Reference level for education level is less than bachelor’s degree. The self-perspective and societal perspective responses were dichotomized to willing and not willing (reference level is ‘not willing’). OR = adjusted odds ratios.

## Discussion

There is a longstanding concern about the accuracy of surrogate consent in representing the wishes of those who lose their ability to decide for themselves [7], [9], [10]. But there is also evidence, both in treatment [12], [13], [14] and research contexts [4], [5], [11], [15], [16], that people are willing to grant their future surrogates leeway, even to the point of surrogates going against their currently stated wishes. Given that the concept of leeway challenges the traditional focus on honoring stated preferences, it seems important to examine the validity of leeway preferences, especially for surrogate consent for research that has complex scientific, legal, historical, and bioethical dimensions. We characterized the effect of democratic deliberation on this willingness to grant leeway, and also compared subjects’ responses given as potential future research subjects with their responses given as surrogates for their loved ones.

We found that the DD group was more willing to give leeway than controls for the lumbar puncture scenario (both immediately after DD session and a month later), vaccine scenario (after DD session but not sustained), and gene transfer scenario (one month after DD session). Even for a high risk scenario such as gene transfer, 71% were willing to give leeway to their future surrogates a month after the DD session. These findings validate the overall high rates of willingness to grant leeway found in traditional surveys. The willingness to grant leeway to one’s future surrogates appears to be a fairly robust phenomenon that needs to be incorporated in debates about ethics of surrogate consent for research. In particular, eliciting subject preferences regarding participation in future dementia research, whether as part of a research study (such as our study) or as part of a clinic’s practice in eliciting advance preferences, should include questions about leeway.

There were other notable findings about leeway preferences in our study. At baseline prior to the deliberation session, a majority of respondents (57%–71%, depending on scenario) were willing to give some or complete leeway to a close family member to go against their currently stated preferences about future dementia research participation. This is consistent with prior studies which found that senior center attendees were willing to grant leeway at rates of 70% (lumbar puncture) and 81% (blood draw) [16], and a national survey of older Americans using the same four scenarios that found 55% to 67% of respondents willing to grant some or complete leeway [5].

In terms of predictors of leeway responses, demographic variables in general were not robust predictors. However, willingness to volunteer for future dementia research and support for a societal policy allowing familial surrogate consent for dementia research were the strongest predictors of willingness to give leeway. This finding is consistent with our previous national survey study [5], and may reflect recognition on the part of people willing to participate in research that there might be circumstances in which a surrogate would deem enrollment unwise, and hence they would allow a surrogate to act in a manner thought to be in their best interests.

Related to the previous point, it is notable that even among respondents who stated that they were *not* willing to participate in future dementia research at a point when they had lost capacity, a significant minority (36–43% depending on scenario) stated that they would be willing to give leeway to a surrogate decision-maker to go against their currently stated preference. Taking this into account (i.e., adding those who are willing to participate with those who are not willing but are willing to grant leeway), the proportion of potential research enrollees become significantly larger (66–95% depending on scenario). This was most notable for the two higher-risk scenarios of vaccine testing and gene transfer study. For example, 47.5% said they were willing to participate in the gene transfer scenario; an additional 18.5% said no, but were willing to give leeway. This differential effect on higher risk scenarios may reflect the fact that the lower risk scenarios tended to have a ceiling effect. Further, content analysis of subjects’ comments shows that among those respondents who were willing to give leeway, an important reason was that surrogates may have more or better information in the future, implying both trust in their surrogates as well as recognition of the uncertainties of present preferences about future decisions. On the other hand, those who were not willing to give leeway often saw the possibility of going against their stated wishes as intrinsically wrong. Thus, they considered the question of whether to allow leeway mostly as a matter of principle rather than whether it would be desirable for surrogates to have the flexibility to respond to situations that cannot be anticipated at the time when an advance decision is made.

We also found that the in-depth education and peer deliberation involved in democratic deliberation had significant effects on respondents’ willingness to participate in future dementia research. The deliberation group became more willing than controls to participate in all scenarios after deliberation, and this change was sustained even after 1 month. Further, for all scenarios, respondents were more willing to enroll *themselves* in research than to enroll loved ones, suggesting a tendency to act in a protective fashion as a surrogate.

The study has several limitations. First, among respondents willing to grant leeway, there were many more respondents who would grant only some leeway over complete leeway. As we did not define “some leeway” or “complete leeway,” we need to be cautious in our interpretation. Future research should attempt to clarify the difference between the two response categories. Second, although the internal validity of the study was high, external validity may be limited by the considerable time commitment required from volunteer participants for the deliberation sessions and the consequent likelihood of self-selection. However, our baseline results were similar to those found in a previous cross-sectional study of the older general public (≥50 years old), providing some evidence that this group may not differ in substantive attitudes toward surrogate consent compared to the national sample [5]. Third, there may have been undetected group dynamics or subtle influences from the experts during the deliberation session that affected the deliberations. However, careful qualitative analyses of the sessions show that the quality of deliberation was quite high and that participants were very satisfied with the sessions, learned and used new information, were respectful and collaborative, and were able to “reason together” effectively [18]. Finally, because the attitudes elicited were based upon information unique to dementia research, findings may not be generalizable to other research, e.g., research involving comatose subjects or incapacitated persons with mental illness.

In conclusion, most people in our study (like those in previous studies of caregivers, [4] senior center attendees, [16] and the older general public) were willing to grant leeway regarding choices about research participation to their future surrogates–even when leeway is explicitly defined as going against their stated preferences. Further, this willingness is not an artifact of lack of knowledge or deliberation, since in-depth education with peer deliberation in fact tend to increase their willingness to grant such leeway. It is also notable that a significant minority (36%–43%) of our participants who said they would not want to volunteer for research in case of future incapacity nonetheless was willing to grant leeway. These results strongly suggest that a person’s current preferences about future research participation are not the only–or depending on the circumstances, even the most important–legitimate basis for his or her future surrogate’s decisions. Although a conventional bioethics framework may favor eliciting and relying on statements of future preference [19], it appears that most people are aware of future uncertainties and are open to the idea that their surrogates might be in a better position to make those decisions than they themselves are today.

## Supporting Information

Figure S1 **Baseline (Survey 1) responses of the overall study sample, regarding willingness to participate in future research (self-perspective), willingness to provide leeway to surrogates (leeway), and willingness to enroll a loved one in research (surrogate perspective). N = 503.**
(PDF)

Table S1 **Distribution of willingness to participate in research (self-perspective) and to grant leeway to family member in making research decisions if respondent is unable to make decision for self, measured at three time points.**
(PDF)

Table S2 **Distribution of willingness to give permission for a loved one to participate in research (surrogate perspective), by study arm, measured three times.**
(PDF)
