# Supplementary material for: How Important Is ‘Accuracy’ of Surrogate Decision-Making for Research Participation?
Source: PLoS One. 2013 Jan 31;8(1):e54790. doi: 10.1371/journal.pone.0054790 (PMC3561414; doi:10.1371/journal.pone.0054790)
Supplement: Figure S1 — Baseline (Survey 1) responses of the overall study sample, regarding willingness to participate in future research (self-perspective), willingness to provide leeway to surrogates (leeway), and willingness to enroll a loved one in research (surrogate perspective). N = 503. (PDF) [file pone.0054790.s001.pdf]

Figure S1. Baseline (Survey 1) responses of the overall study sample, regarding willingness to participate in future research (self-perspective), willingness to provide leeway to surrogates (leeway), and willingness to enroll a loved one in research (surrogate perspective). N=503.

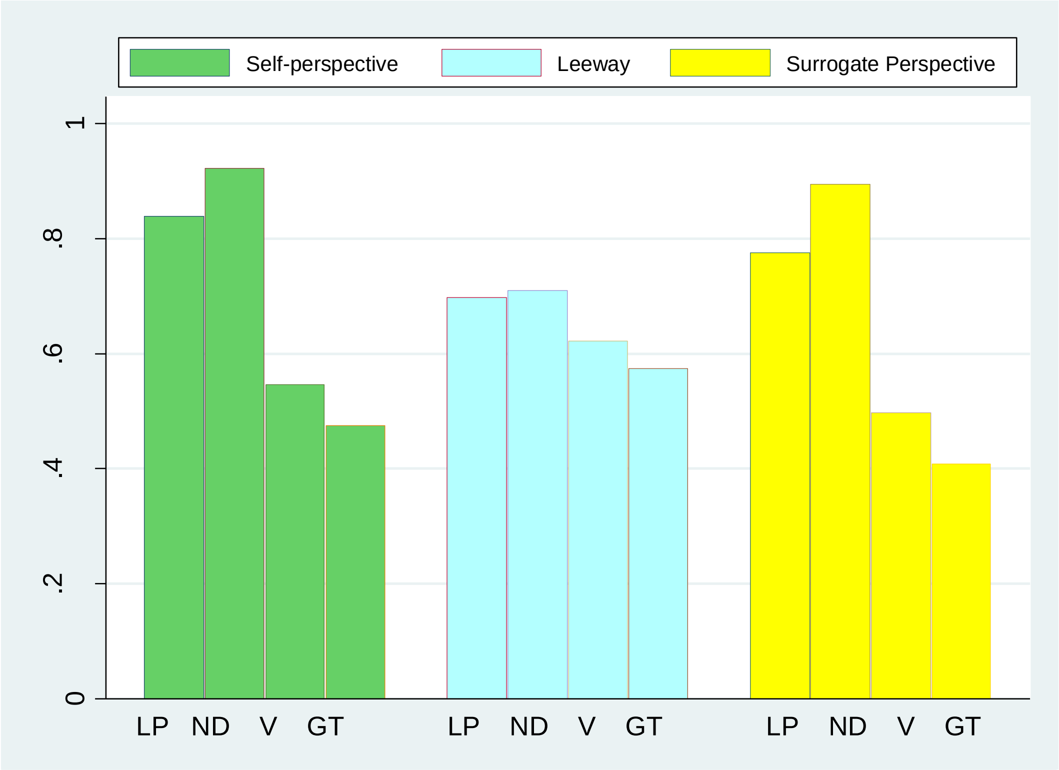

LP=lumbar puncture, ND=new drug RCT, V=vaccine RCT, GT=gene transfer. Willingness includes both ‘probably’ and ‘definitely’ willing for the self-perspective and surrogate perspective responses and ‘some’ and ‘complete’ leeway for the leeway response.
