# Supplementary material for: How Important Is ‘Accuracy’ of Surrogate Decision-Making for Research Participation?
Source: PLoS One. 2013 Jan 31;8(1):e54790. doi: 10.1371/journal.pone.0054790 (PMC3561414; doi:10.1371/journal.pone.0054790)
Supplement: Table S1 — Distribution of willingness to participate in research (self-perspective) and to grant leeway to family member in making research decisions if respondent is unable to make decision for self, measured at three time points. (PDF) [file pone.0054790.s002.pdf]

	**Table S1**. Distribution of willingness to participate in research (self-perspective) and to grant leeway to family member in making research decisions if respondent is unable to make decision for self, measured at three time points.														
	DD Group					Education Group					Control Group				
	Survey 1 (n=212)	Survey 2 (n=173)	Survey 3 (n=168)	Survey 1v2	Survey 1v3	Survey 1 (n=141)	Survey 2 (n=129)	Survey 3 (n=128)	Survey 1v2	Survey 1v3	Survey 1 (n=150)	Survey 2 (n=140)	Survey 3 (n=143)	Survey 1v2	Survey 1v3
Lumbar Puncture	%	%	%	p-value	p-value	%	%	%	p-value	p-value	%	%	%	p-value	p-value
Definitely Not	6.1	2.3	0.6	0.001	0.092	7.8	4.7	8.6	0.558	0.395	2.7	2.9	2.1	0.666	0.552
Probably Not	8.0	4.0	9.5			11.3	14.0	12.5			12.7	10.0	16.8		
Probably Yes	41.5	30.6	36.3			39.7	43.4	45.3			46.0	53.6	48.3		
Definitely Yes	43.9	62.4	53.0			40.4	37.2	33.6			38.0	32.9	30.8		
No Leeway	30.2	23.1	23.8	0.147	0.331	30.5	29.5	35.9	0.385	0.514	28.7	29.3	30.1	0.682	0.537
Some Leeway	53.3	54.9	58.9			54.6	51.2	49.2			56.0	57.9	57.3		
Complete Leeway	14.6	20.8	17.3			14.2	18.6	14.8			14.7	11.4	9.8		
New Drug RCT	%	%	%	p-value	p-value	%	%	%	p-value	p-value	%	%	%	p-value	p-value
Definitely Not	1.9	1.2	0.6	0.250	0.554	2.8	3.9	4.7	0.379	0.058	3.3	3.6	0.7	0.793	0.257
Probably Not	2.4	2.3	3.0			5.0	7.0	7.8			8.7	9.3	9.1		
Probably Yes	40.6	31.8	36.3			43.3	48.1	50.0			48.0	47.9	55.2		
Definitely Yes	55.2	64.7	59.5			48.9	40.3	37.5			40.0	39.3	34.3		
No Leeway	27.4	25.4	23.8	0.554	0.172	30.5	28.7	29.7	0.387	0.443	29.3	22.9	25.2	0.390	0.771
Some Leeway	54.7	54.3	56.5			53.2	49.6	54.7			57.3	64.3	61.5		
Complete Leeway	17.0	20.2	19.6			16.3	21.7	15.6			12.7	12.9	11.9		
Vaccine	%	%	%	p-value	p-value	%	%	%	p-value	p-value	%	%	%	p-value	p-value
Definitely Not	12.7	10.4	6.0	0.006	0.005	20.6	18.6	20.3	0.470	0.923	15.3	17.1	9.8	0.162	0.087
Probably Not	29.7	19.1	20.8			27.7	20.9	24.2			30.0	26.4	30.1		
Probably Yes	38.7	40.5	44.6			32.6	38.8	37.5			42.0	39.3	43.4		
Definitely Yes	18.4	29.5	27.4			19.1	20.9	18.0			12.0	15.7	16.8		
No Leeway	35.8	31.2	23.2	0.003	0.007	41.1	35.7	42.2	0.649	0.490	36.0	39.3	29.4	0.092	0.152
Some Leeway	53.8	48.6	61.3			47.5	48.8	42.2			54.7	48.6	57.3		
Complete Leeway	8.5	19.7	14.9			11.3	15.5	15.6			8.0	10.7	11.9		
Gene Transfer	%	%	%	p-value	p-value	%	%	%	p-value	p-value	%	%	%	p-value	p-value
Definitely Not	17.9	20.2	17.3	0.005	0.005	28.4	27.1	28.9	0.062	0.397	22.7	27.1	23.1	0.794	0.857
Probably Not	31.1	26.0	22.6			29.1	20.2	25.0			29.3	27.9	30.1		
Probably Yes	40.1	32.9	37.5			27.7	38.0	33.6			36.7	35.7	35.7		
Definitely Yes	10.8	20.8	22.0			14.9	14.7	12.5			10.7	9.3	11.2		
No Leeway	36.8	38.7	28.6	0.074	0.013	48.2	45.7	46.1	0.791	0.855	45.3	47.1	43.4	0.387	0.835
Some Leeway	52.4	45.7	51.8			41.1	40.3	39.1			46.0	40.7	46.2		
Complete Leeway	10.4	15.0	19.6			10.6	14.0	14.1			8.0	11.4	9.8		

The p-values are based on asymptotic symmetry test for paired data, each with multiple discrete response levels (STATA command, “symmetry”). Some percentages do not add to 100 because not all participants answered the question.
