# Supplementary material for: How Important Is ‘Accuracy’ of Surrogate Decision-Making for Research Participation?
Source: PLoS One. 2013 Jan 31;8(1):e54790. doi: 10.1371/journal.pone.0054790 (PMC3561414; doi:10.1371/journal.pone.0054790)
Supplement: Table S2 — Distribution of willingness to give permission for a loved one to participate in research (surrogate perspective), by study arm, measured three times. (PDF) [file pone.0054790.s003.pdf]

Table S2. Distribution of willingness to give permission for a loved one to participate in research (surrogate perspective), by study arm, measured at three time points.

Research Scenario	DD Group					Education Group					Control Group				
	Survey 1 (n=212)	Survey 2 (n=173)	Survey 3 (n=168)	Survey 1v2	Survey 1v3	Survey 1 (n=141)	Survey 2 (n=129)	Survey 3 (n=128)	Survey 1v2	Survey 1v3	Survey 1 (n=150)	Survey 2 (n=140)	Survey 3 (n=143)	Survey 1v2	Survey 1v3
Lumbar Puncture	%	%	%	p-value	p-value	%	%	%	p-value	p-value	%	%	%	p-value	p-value
Definitely Not	8.0	1.2	3.0	0.000	0.060	7.8	7.0	10.2	0.309	0.461	7.3	7.1	5.6	0.667	0.387
Probably Not	11.8	8.1	9.5			18.4	15.5	19.5			14.0	19.3	20.3		
Probably Yes	57.1	48.0	61.3			56.0	56.6	52.3			56.0	52.1	49.7		
Definitely Yes	22.6	42.2	26.2			17.7	20.2	17.2			22.0	20.0	20.3		
New Drug RCT	%	%	%	p-value	p-value	%	%	%	p-value	p-value	%	%	%	p-value	p-value
Definitely Not	1.4	1.7	1.8	0.421	0.884	3.5	4.7	4.7	0.932	0.619	4.7	5.7	2.8	0.460	0.370
Probably Not	4.7	3.5	4.2			10.6	10.1	12.5			8.0	10.0	11.2		
Probably Yes	59.9	49.7	59.5			58.9	59.7	59.4			56.7	60.7	60.1		
Definitely Yes	34.0	45.1	34.5			26.2	25.6	22.7			30.0	22.1	23.1		
Vaccine	%	%	%	p-value	p-value	%	%	%	p-value	p-value	%	%	%	p-value	p-value
Definitely Not	12.7	11.0	8.3	0.017	0.002	19.9	17.8	25.0	0.109	0.011	14.7	21.4	14.7	0.409	0.973
Probably Not	34.9	24.9	27.4			32.6	24.0	22.7			34.0	31.4	31.5		
Probably Yes	42.5	46.8	50.6			34.0	45.7	43.8			42.7	39.3	46.2		
Definitely Yes	9.4	16.8	13.7			11.3	10.9	8.6			6.7	6.4	7.0		
Gene Transfer	%	%	%	p-value	p-value	%	%	%	p-value	p-value	%	%	%	p-value	p-value
Definitely Not	21.7	26.6	18.5	0.009	0.247	27.7	27.1	28.9	0.005	0.524	25.3	33.6	30.1	0.266	0.449
Probably Not	33.5	25.4	30.4			37.6	25.6	31.3			31.3	28.6	29.4		
Probably Yes	37.7	37.0	39.9			29.1	38.8	33.6			38.0	32.9	35.0		
Definitely Yes	5.7	11.0	10.7			4.3	8.5	5.5			4.7	5.0	4.9		

The p-values are based on asymptotic symmetry test for paired data, each with multiple discrete response levels (STATA command, "symmetry"). Some percentages do not add to 100 because not all participants answered the question.
